# Community-acquired multidrug-resistant *Enterobacter cloacae* sepsis in a 25-month-old child in rural Gambia: A case report

**DOI:** 10.1016/j.idcr.2024.e02018

**Published:** 2024-06-27

**Authors:** Baleng Mahama Wutor, Williams Oluwatosin Adefila, Keita Modou Lamin, Yusuf Abdulsalam O, Ilias Hossain, Minteh Molfa, Ousman Barjo, Rasheed Salaudeen, Isaac Osei, Grant Mackenzie

**Affiliations:** a Medical Research Council Unit The Gambia at London School of Hygiene & Tropical Medicine, Fajara, The Gambia; b Faculty of Infectious and Tropical Diseases, London School of Hygiene & Tropical Medicine, London, UK; c Murdoch Children’s Research Institute, Melbourne, Australia; d Department of Paediatrics, University of Melbourne, Australia

**Keywords:** Case report, *Enterobacter cloacae*, *Enterobacter sepsis*, Multidrug-resistant, Community-acquired, Mortality

## Abstract

*Enterobacter cloacae* is the leading cause of morbidity and mortality in the genus *Enterobacter*. It mostly causes nosocomial infections, especially in children, the elderly and those with underlying diseases. However, cases of community-acquired bacteraemia caused by *E. cloacae* have been reported. The increasing inclination of *E. cloacae* to cause multidrug-resistant infections has made it particularly challenging to treat. A 25-month-old male child presented to a rural hospital in The Gambia with a one-week history of persistent high-grade fever, dyspnoea, and anorexia. Two days before presentation, he began to have generalized tonic-clonic seizures. On examination, he was found to be febrile, dyspnoeic, pale, and tachycardic. He had a modified Glasgow Coma Scale score of 9/15. Investigations revealed an elevated C-reactive protein, low haemoglobin, and elevated white blood cell count. Cerebrospinal fluid culture did not yield any growth. *E. cloacae* was isolated from a blood culture taken on the day of admission. The pathogen was resistant to all available antibiotics. He was transfused with whole blood and initially treated empirically with amoxicillin-clavulanic acid and gentamicin. The former was changed to cefuroxime because the child had not improved. The child died nine days after admission. Although *E. cloacae* is primarily known for causing nosocomial infections, fatal community-acquired infections also occur. This case report demonstrates the difficulty in treating multidrug-resistant *E. cloacae* in a low-resource setting and its propensity to cause fatal infections.

## Background

*Enterobacter cloacae* is a gram-negative anaerobic bacterium that belongs to the genus *Enterobacter* and the family *Enterobacteriaceae* [1]. Some of the organisms in this genus, such as *Enterobacter aerogenes* (reclassified as *Klebsiella aerogenes*) and *E. cloacae* cause considerable opportunistic infections in immunocompromised and hospitalized patients [2]. The family *Enterobacteriaceae* has been designated by the World Health Organization (WHO) as one of the most critical groups of antibiotic-resistant bacteria [3]. Of the organisms in the genus *Enterobacter*, *E. cloacae* causes the most morbidity and mortality [4].

*E. cloacae* has been increasingly recognized as a significant nosocomial pathogen [2]. However, community-acquired *E. cloacae* bacteraemia has also been reported [5]. Beyond the increasing burden of infections caused by *E. cloacae*, its intrinsic resistance to β-lactam antibiotics and cephalosporins is of concern [1]. It has the propensity to cause outbreaks, especially in paediatric units [6].

Treatment of *E. cloacae* bacteraemia is complicated by the broad resistance it demonstrates towards commonly used antibiotics [7]. As a result of the extended-spectrum β-lactamase (ESBL) genes that render *E. cloacae* resistant to β-lactam and cephalosporin antibiotics, some case reports have shown success in using carbapenems (imipenem and meropenem) and aminoglycosides (amikacin and gentamicin) for the treatment of serious infections caused by *E. cloacae* [8]. However, carbapenemase-producing *E. cloacae* are also being increasingly reported [9].

Most *Enterobacter spp.* isolated from blood cultures are multidrug-resistant (defined as acquired resistance to at least one agent in three or more antimicrobial categories) [10], [11]. The prevalence of *Enterobacter spp.* resistance to cephalosporins is as high as 40 % and resistance to aminoglycosides ranges from 10–50 % [12], [13]. Case fatality associated with infections due to multidrug-resistant *E. spp.* infections range between 18–32 %, and nosocomial infections generally have worse outcomes than community-acquired infections [14]. Factors associated with deaths that are attributable to invasive *E. cloacae* include low haemoglobin levels, elevated C-reactive protein, respiratory failure, renal failure, and septic shock [15].

*E. cloacae* sepsis is not only common in low-resource settings, but is particularly difficult to treat due to high resistance and the limited repertoire of antibiotics in these settings [16]. Isolated pathogens are often found to be resistant to available antibiotics.

In this case report, we describe the clinical course and unsuccessful treatment of a 25-month-old child who had multidrug-resistant bacteraemic *E. cloacae* sepsis.

## Case presentation

The Gambia is a small country in West Africa with a population of approximately 2.7 million people and a Gross Domestic Product per capita of $ 808 [17]. The country has a coastline on the Atlantic Ocean and extends approximately 400 km from west to east into the interior rural regions. The public health system operates with limited human and physical resources.

A 25-month-old male child who had no known underlying disease presented to a hospital in eastern Gambia with a one-week history of persistent high-grade fever, dyspnoea, and anorexia. Two days before presentation, he began to have multiple generalized tonic—clonic seizures, each lasting for approximately a minute with associated upward eye-rolling, tongue biting, and bleeding from the mouth. On the day of presentation, the child had become unconscious after an episode of seizure and was rushed to the health facility. The infant welfare card was not available to confirm the vaccines the child had received. His parents, however, asserted that he had received some of the required vaccinations.

On examination, he had a temperature of 39.9 °C, respiratory rate of 60 cycles/min, oxygen saturation of 89 %, pulse of 170 beats/min, and a Modified Glasgow Coma Scale score of 9/15. He had conjunctival pallor but was not jaundiced. He had lower chest wall indrawing and flaring of the alae nasi. On auscultation, the breath sounds were broncho-vesicular with no crepitations or rhonchi. The heart sounds were normal. There was no nuchal rigidity, and both Kernig’s and Brudzinski’s signs were negative. His pupils reacted poorly to light. The rest of the physical examination was normal.

The investigations undertaken are shown in Table 1. Notably, the child had severe anaemia (Hb of 5.5 g/dL), an elevated white blood cell count, elevated C-reactive protein and random blood glucose on admission ranged between 9.5 mmol/L and 11.5 mmol/L. *E. cloacae* was isolated from the blood using standard microbiological procedures. After incubation in MacConkey agar, mucoid lactose fermenting colonies were identified. Gram staining revealed gram-negative rods. The oxidase test was negative. The bacterium was then processed for identification using API 20E (Analytic Profile Index 20E). The API indicated an identification number of 6305573, consistent with *E. cloacae.* Antibiotic susceptibility testing was performed and interpreted using the standard disc diffusion method [18]. The pathogen was resistant to all available antibiotics, it was susceptible to kanamycin and mecillinam.

**Table 1:** Results of investigations.

**Investigation**	**Result**		**Reference range**
C-reactive protein	48 mg/L		0 – 10 mg/L
Malaria Rapid Diagnostic Test	Negative		
**Full Blood Count**	**Blood cell**	**Cell count**	**Reference range**
	Red blood cell count	3.76* 10^12/l	3.50 – 5.20* 10^12/l
	Haemoglobin	5.5 g/dL	12.0 –16.0 g/dL
	MCV	52.7 fL	80.0 –100.0 fL
	MCH	14.6 pg	27.0 – 34.0 pg
	MCHC	27.7 g/dL	31.0 – 37.0 g/dL
	White blood cell count	22.72* 10^9/L	4.00 – 12.00* 10^9/L
	Granulocyte count	18.86* 10^9/L	2.00 – 7.00* 10^9/L
	Platelets	346* 10^9/L	100 – 300* 10^9/L
	Lymphocyte count	2.41* 10^9/L	0.80 – 7.00* 10^9/L
**Cerebrospinal fluid analysis**	**Parameter**	**Result**	
	Leucocytes	Negative	
	Nitrogen	Negative	
	Erythrocytes	Negative	
	Glucose	250 mg/dL	50 – 80 mg/dL
	Protein	30 mg/dL	15 – 45 mg/dL
**Cerebrospinal fluid culture**	No growth		
**Isolate from Blood culture**	*Enterobacter cloacae*		
**Blood culture susceptibility results**	**Antibiotic**	**Diameter of zone of inhibition**	Susceptibility interpretation*
	ceftriaxone	8	Resistant
	ampicillin	0	Resistant
	amoxicillin-clavulanic acid	0	Resistant
	chloramphenicol	0	Resistant
	tetracycline	0	Resistant
	cotrimoxazole	0	Resistant
	cefotaxime	0	Resistant
	ceftazidime	17	Resistant
	gentamicin	12	Resistant
	cefoxitin	0	Resistant
	ciprofloxacin	0	Resistant
	penicillin	0	Resistant
	novobiocin	0	Resistant
	colistin sulphate	0	Resistant
	oxacillin	0	Resistant
	nalidixic acid	0	Resistant
	clindamycin	0	Resistant
	azithromycin	0	Resistant
	mecillinam	18	Susceptible
	kanamycin	16	Susceptible

* Interpretations using European Committee on Antibiotic Susceptibility Testing breakpoints [18]

A diagnosis of sepsis with probable meningitis and severe anaemia was made. The child was treated empirically with intravenous amoxicillin-clavulanic acid 45 mg/kg twelve hourly, intravenous gentamicin 3 mg /kg daily, intravenous phenytoin 20 mg/kg stat, then 5 mg/kg twelve hourly, intravenous dextrose saline and supplemental oxygen via nasal prongs at 2 L/min. He was transfused with 20 ml/kg of whole blood. Although the antibiotic susceptibility results were available by the third day of admission and there was demonstrated susceptibility to mecillinam and kanamycin, both were not available. On the third day of admission, amoxicillin-clavulanic acid was replaced with intravenous cefuroxime 50 mg/kg twice daily, however, his condition did not improve. He died after nine days of admission.

## Discussion

*E. cloacae* mostly cause nosocomial infections and invasive disease in people with underlying comorbidities. Risk factors such as extremes of age, prior use of antibiotics, and invasive procedures have also been associated with *E. cloacae* infection. Multidrug-resistant *E. cloacae* is a risk factor for mortality, with case fatality as high as 33.7 % [11].

In recent years, there have been increasing reports of multidrug-resistant *Enterobacteriaceae* in sub-Saharan Africa. Sanneh B et al. found a high prevalence of multidrug-resistant *Enterobacteriaceae* in the stool of food handlers in The Gambia [19]. *Enterobacter spp.* were the commonest *Enterobacteriaceae* isolated in the study. Secka F et al., in a prospective hospital-based study that investigated children presenting to two urban hospitals in The Gambia also found a high prevalence of gram-negative isolates that were resistant to both first and second-line antibiotics (39 % and 46 %, respectively) [20]. With the proliferation of pharmacies across the country, including in rural communities, the over-the-counter dispensing of antibiotics has created an avenue for bacteria to develop resistance. Such multidrug-resistant bacteria can then be transmitted through faecal-oral and other routes.

Our patient had no history of previous hospitalization before presenting to the hospital. He also had no known underlying condition or immunodeficiency. Notwithstanding, the paediatric population is particularly vulnerable to infections such as *E. cloacae*. Liu et al. found that children under 9 years and adults above 60 years of age are most at risk of *E. cloacae* bacteraemia [21]. Additionally, our patient had severe anaemia that required a blood transfusion and an elevated C-reactive protein. Both have been identified as factors associated with deaths attributable to invasive *E. cloacae* disease [15].

The *E. cloacae* isolated from our patient far exceeded the minimum threshold for describing *Enterobacteriaceae* as multidrug-resistant [10]. In fact, it was resistant to at least one agent in as many as nine antimicrobial groups. Although the culture results demonstrated sensitivity to kanamycin and mecillinam, neither was available. Most health facilities in The Gambia have limited antibiotics. The first-line antibiotics for most conditions (ampicillin and gentamicin) are usually available. Second-line antibiotics such as ceftriaxone are sometimes available. Monobactams and carbapenems are not routinely available in rural health facilities (including ours), and their cost prohibits their use even when indicated. The challenge in obtaining the appropriate antibiotics in our facility may have contributed to the death of our patient.

In low-resource settings such as ours, it is difficult to diagnose and treat gram-negative sepsis. This is because blood cultures and susceptibility testing are not routinely done and there is a limited number of antibiotics available.

Carbapenem and monobactam susceptibilities were not tested because they were unavailable. Also, being a case report, our findings cannot be generalized. Even though this case report may not be directly relevant for clinical decision-making, it highlights an important growing trend of community-acquired *E. cloacae* infections and their propensity to be multidrug-resistant. It also highlights the importance of aggressively treating these infections because of their associated high morbidity and mortality.

## Conclusion

Although *E. cloacae* mostly causes nosocomial infections, community-acquired infections also occur and can be as fatal as nosocomial infections. The inclination of *E. cloacae* to be multidrug-resistant makes it challenging to achieve successful treatment. This case report demonstrates the unsuccessful treatment of sepsis resulting from multidrug-resistant *E. cloacae* infection in a 25-month-old male child.

## Author contributions

Dr Baleng Mahama Wutor and Dr Williams Oluwatosin Adefila wrote the initial draft. All authors reviewed and contributed to the manuscript.

## Consent

Written informed consent was obtained from the parents of the patient for the publication of this case report. A copy of the written consent is available for review by the Editor-in-Chief of this journal.

## Funding

This research did not receive any specific grant from funding agencies in the public, commercial, or not-for-profit sectors.

## CRediT authorship contribution statement

**Ousman Barjo:** Writing – review & editing, Investigation, Formal analysis. **Rasheed Salaudeen:** Writing – review & editing, Investigation, Formal analysis. **Isaac Osei:** Writing – review & editing, Supervision, Formal analysis, Conceptualization. **Grant Mackenzie:** Writing – review & editing, Supervision, Conceptualization. **Baleng Mahama Wutor:** Writing – review & editing, Writing – original draft, Formal analysis, Data curation, Conceptualization. **Williams Oluwatosin Adefila:** Writing – review & editing, Writing – original draft, Formal analysis, Data curation, Conceptualization. **Keita Modou Lamin:** Writing – review & editing, Formal analysis, Data curation, Conceptualization. **Yusuf Abdulsalam O.:** Writing – review & editing, Formal analysis. **Hossain M Ilias:** Writing – review & editing, Formal analysis. **Minteh Molfa:** Writing – review & editing, Investigation, Formal analysis, Data curation.

## Declaration of Competing Interest

The authors declare that they have no known competing financial interests or personal relationships that could have appeared to influence the work reported in this paper.
